# Endoplasmic reticulum stress in spinal and bulbar muscular atrophy: a potential target for therapy

**DOI:** 10.1093/brain/awu114

**Published:** 2014-06-04

**Authors:** Karli Montague, Bilal Malik, Anna L. Gray, Albert R. La Spada, Michael G. Hanna, Gyorgy Szabadkai, Linda Greensmith

**Affiliations:** 1 Sobell Department of Motor Neuroscience and Movement Disorders, Institute of Neurology, UCL, Queen Square, London, WC1N 3BG, UK; 2 MRC Centre for Neuromuscular Diseases, Institute of Neurology, UCL, Queen Square, London, WC1N 3BG, UK; 3 Department of Paediatrics, University of California San Diego, La Jolla, CA 92093, USA; 4 Department of Cellular & Molecular Medicine and Neurosciences, University of California San Diego, La Jolla, CA 92093, USA; 5 Division of Biological Sciences, University of California San Diego, La Jolla, CA 92093, USA; 6 Institute for Genomic Medicine, University of California San Diego, La Jolla, CA 92093, USA; 7 Sanford Consortium for Regenerative Medicine, University of California San Diego, La Jolla, CA 92093, USA; 8 Rady Children’s Hospital, San Diego, CA 92123, USA; 9 Department of Molecular Neuroscience, Institute of Neurology, UCL, Queen Square, London, WC1N 3BG, UK; 10 Cell and Developmental Biology Department, UCL, Gower Street, London, WC1E 6BT, UK; 11 Department of Biomedical Sciences, University of Padua and CNR Neuroscience Institute, Padua, Italy

**Keywords:** endoplasmic reticulum stress, polyglutamine expansions, motor neuron disease, calcium, SBMA

## Abstract

Spinal and bulbar muscular atrophy is an X-linked degenerative motor neuron disease caused by an abnormal expansion in the polyglutamine encoding CAG repeat of the androgen receptor gene. There is evidence implicating endoplasmic reticulum stress in the development and progression of neurodegenerative disease, including polyglutamine disorders such as Huntington’s disease and in motor neuron disease, where cellular stress disrupts functioning of the endoplasmic reticulum, leading to induction of the unfolded protein response. We examined whether endoplasmic reticulum stress is also involved in the pathogenesis of spinal and bulbar muscular atrophy. Spinal and bulbar muscular atrophy mice that carry 100 pathogenic polyglutamine repeats in the androgen receptor, and develop a late-onset neuromuscular phenotype with motor neuron degeneration, were studied. We observed a disturbance in endoplasmic reticulum-associated calcium homeostasis in cultured embryonic motor neurons from spinal and bulbar muscular atrophy mice, which was accompanied by increased endoplasmic reticulum stress. Furthermore, pharmacological inhibition of endoplasmic reticulum stress reduced the endoplasmic reticulum-associated cell death pathway. Examination of spinal cord motor neurons of pathogenic mice at different disease stages revealed elevated expression of markers for endoplasmic reticulum stress, confirming an increase in this stress response *in vivo*. Importantly, the most significant increase was detected presymptomatically, suggesting that endoplasmic reticulum stress may play an early and possibly causal role in disease pathogenesis. Our results therefore indicate that the endoplasmic reticulum stress pathway could potentially be a therapeutic target for spinal and bulbar muscular atrophy and related polyglutamine diseases.

## Abstract (precis)

Spinal and bulbar muscular atrophy is a degenerative motor neuron disease caused by CAG repeat expansion in the androgen receptor gene. Montague *et al*. reveal an early increase in endoplasmic reticulum stress in a mouse model, and suggest that this pathway may be a therapeutic target for polyglutamine diseases.

## Introduction

Spinal and bulbar muscular atrophy (SBMA), also known as Kennedy’s disease, is an X-linked degenerative motor neuron disease caused by a polyglutamine encoding CAG repeat expansion in exon 1 of the androgen receptor gene (La Spada *et al.*, 1991). SBMA belongs to a family of nine polyglutamine repeat expansion diseases including Huntington's disease, dentatorubral pallidoluysian atrophy and six spinocerebellar ataxias, which share many common features and underlying pathological mechanisms (Ross, 2002; Gatchel and Zoghbi, 2005). In SBMA, the polymorphic trinucleotide CAG repeat normally ranges from 9 to 35, but an expansion of >37 repeats results in disease (Fischbeck, 2001; Rocchi and Pennuto, 2013). Symptoms resulting from loss of lower motor neurons in the spinal cord and brainstem manifest primarily in males between 30 and 50 years of age and include proximal limb and bulbar muscle weakness, atrophy and fasciculations (Katsuno *et al.*, 2012; Rocchi and Pennuto, 2013). Endocrine abnormalities may also be present, with signs of gynaecomastia, testicular atrophy and reduced fertility.

The androgen receptor is a steroid hormone receptor held in the cytoplasm in a complex with heat shock proteins Hsp90 and Hsp70. Binding of androgen ligand allows the androgen receptor to dissociate from the complex, permitting it to translocate to the nucleus, resulting in activation of target genes (Heinlein and Chang, 2001). However, expansion of the polyglutamine repeat tract in the androgen receptor results in a pathogenic conformational change in the androgen receptor protein, facilitating its aggregation into inclusions (Perutz *et al.*, 1994; Williams and Paulson, 2008). Although the precise mechanisms involved in the death of motor neurons in SBMA are unknown, the endoplasmic reticulum stress response has been shown to play a role in the pathogenesis of other motor neuron disorders, such as amyotrophic lateral sclerosis (Saxena *et al.*, 2009) as well as polyglutamine diseases such as Huntington’s disease (Stenoien *et al.*, 1999; Duennwald and Lindquist, 2008; Reijonen *et al.*, 2008).

**Figure 1:**
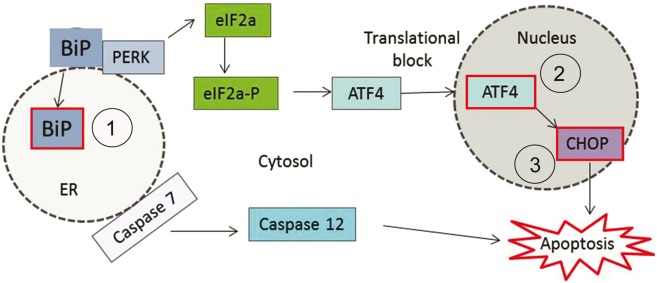
The unfolded protein response illustrating the activation of PERK and increase of binding immunoglobulin protein (BiP), EIF2A-P, ATF4 and CHOP. One arm of the unfolded protein response, which appears to be elevated in motor neuron disease, is initiated by the activation of PERK. The schematic depicts this pathway and highlights the markers that are examined in this study (red boxes). They included: (1) BiP, a misfolded protein sensor; (2) ATF4, a transcription factor that is exempt from the global translational arrest that results is a consequence of the unfolded protein response; and (3) CHOP, a pro-apoptotic marker that is transcribed downstream of ATF4. ER = endoplasmic reticulum.

The endoplasmic reticulum is a point of protein ‘quality control’ in cells (Kaufman, 1999) and is involved in several cellular functions including protein folding and Ca^2+^ homeostasis, serving to process nascent membrane and secretory proteins in a Ca^2+^-dependent manner. Depletion of Ca^2+^ jeopardizes normal endoplasmic reticulum functioning and triggers ‘endoplasmic reticulum stress’ (Ma *et al.*, 2002) either due to the accumulation of unfolded or misfolded proteins within the endoplasmic reticulum, or because of Ca^2+^ store depletion (Fig. 1). The resulting cellular mechanisms that attempt to restore endoplasmic reticulum homeostasis include store-operated Ca^2+^ influx, which replenishes Ca^2+^ levels, as well as the unfolded protein response, which alleviates the protein load (Parekh and Penner, 1997; Szegezdi *et al.*, 2006). Evidence has implicated the endoplasmic reticulum protein STIM1 in the mechanism of store-operated Ca^2+^ influx (Smani *et al.*, 2004). STIM1 senses Ca^2+^ depletion and delivers the endoplasmic reticulum to the plasma membrane where it directly activates Orai1 Ca^2+^ influx channels, thereby facilitating endoplasmic reticulum Ca^2+^ reuptake through sarcoendoplasmic reticulum Ca^2+^ ATPases (SERCAs). The unfolded protein response serves to attenuate protein translation and increase the expression of stress response mediators. The most rapidly activated branch of the unfolded protein response is mediated by a protein kinase RNA-like endoplasmic reticulum kinase (PERK), which is activated following dissociation of binding immunoglobulin protein (BiP), an endoplasmic reticulum chaperone that is essential for protein folding (Szegezdi *et al.*, 2006). PERK activation induces phosphorylation of EIF2A, which attenuates translation, thus alleviating protein load (Harding *et al.*, 2000). ATF4, which is exempt from this translational arrest, induces transcription of pro-survival and pro-apoptotic proteins, the balance of which can determine cell survival (Szegezdi *et al.*, 2003, 2006). Pro-survival genes transcribed downstream of ATF4 include BiP, whereas pro-apoptotic genes include CCAAT/enhancer binding protein homologous protein (CHOP, also known as DDIT3). Prolonged endoplasmic reticulum stress renders the unfolded protein response insufficient to restore endoplasmic reticulum homeostasis and induces apoptosis. Caspase 12 is thought to be a key mediator of endoplasmic reticulum stress-induced apoptosis in mice (Yoneda *et al.*, 2001), whereas in human cells either the proapoptotic cascade activated by BIM (also known as BCL2L11) (Soo *et al.*, 2012; Nihei *et al.*, 2013), or Ca^2+^-mediated mitochondrial death has been implicated (Chami *et al.*, 2008). Indeed, caspase 12 knock-out mice demonstrate increased resistance to endoplasmic reticulum stress-induced apoptosis, yet remain vulnerable to other apoptotic mechanisms (Nakagawa and Yuan, 2000).

Despite suggestions that endoplasmic reticulum stress plays a role in the pathogenesis of both motor neuron disease and some polyglutamine repeat disorders, its role in SBMA has not yet been established. Using a mouse model of SBMA that carries a pathogenic 100 glutamine repeat in the androgen receptor (AR100) and which develops a late onset neuromuscular phenotype, accompanied by motor neuron degeneration (Sopher *et al.*, 2004; Malik *et al.*, 2011, 2013) resembling the human disease, we examined the role of endoplasmic reticulum stress in motor neuron death in SBMA. Our results showed that Ca^2+^ depletion within the endoplasmic reticulum and associated endoplasmic reticulum stress occur in cultured embryonic SBMA motor neurons *in vitro*, leading to an activation of the endoplasmic reticulum-associated cell death pathway. Furthermore, markers of endoplasmic reticulum stress were detected in motor neurons from spinal cords of SBMA mice, particularly during very early, presymptomatic stages of the disease. These results suggest that endoplasmic reticulum stress may play an early, causal role in the pathogenesis of SBMA. Importantly, our findings also show that inhibition of endoplasmic reticulum stress *in vitro* with salubrinal significantly reduces the activation of endoplasmic reticulum stress associated apoptosis by supressing the activation of caspase 12. Pharmacological targeting of this cell death pathway may therefore be a potential therapeutic strategy for SBMA, directed at one of the earliest pathological mechanisms observed in SBMA motor neurons to date.

## Materials and methods

### Reagents

Fluo-4 AM, Fura-2 and PCR reagents were obtained from Invitrogen. Salubrinal was obtained from Merck Chemicals. All other reagents were obtained from Sigma-Aldrich.

### Animals

All of the experimental procedures carried out in this study were performed in accordance with the Scientific Procedures Act 1986 under a licence from the UK Home Office and following approval by the Ethical Review Panel of UCL Institute of Neurology. The generation and characterization of the yeast artificial chromosome AR100 mouse model of SBMA has been described previously in detail (Sopher *et al.*, 2004; Thomas *et al.*, 2006; Malik *et al.*, 2011, 2013). The mice were bred and maintained at the UCL Institute of Neurology Biological Services Facility. A colony of AR100 mice was maintained by mating heterozygous AR100 male mice carrying 100 polyglutamine repeats within the androgen receptor (pathogenic mice) with wild-type C57 Black 6J female mice. Genotyping of embryos and adult mice was carried out on tail biopsies and ear punches, respectively. PCR amplification was carried out using a forward and reverse primer (5′-CATCTGAGTCCAGGGGAACAGC-3 and 5′-GCCCAGGCGCTGCCGTAGTCC-3’, respectively) before digestion of the PCR production with the restriction enzyme BglI (New England Biolabs).

### Primary motor neuron cultures

Mixed ventral horn primary motor neuron cultures were prepared from wild-type and AR100 embryonic Day 13 mice as described previously (Malik *et al.*, 2011). Briefly, spinal cords were removed and the ventral horns were isolated and stored in a Hank’s balanced salt solution containing penicillin/streptomycin. Ventral horn tissue samples were dissociated in 0.025% trypsin at 37°C. Motor neurons were plated onto coverslips that had previously been coated with polyornithine (1.5 µg/ml) and laminin (5 µg/ml) for a minimum of 12 and 2 h, respectively. Cultures were maintained until 7 days *in vitro* in Neurobasal® medium that had been supplemented with 50 µ/ml penicillin (Sigma-Aldrich), 2% B27 supplement, 0.05% mercaptoethanol, 2% normal horse serum and 0.5 mM l-glutamine (Invitrogen), 0.1 ng/ml GDNF, 0.1 ng/ml BDNF and 0.5 ng/ml CNTF (Caltag). Cells were maintained at 37°C in 5% CO_2_. For all western blot and immunocytochemistry experiments and for some calcium imaging experiments, cultures were treated for 3 days with 50 nM dihydrotestosterone, the androgen receptor ligand. In some cases cultures were also treated for 24 h with 500 nM salubrinal, an inhibitor of endoplasmic reticulum stress.

Purified motor neuron cultures were prepared from wild-type and AR100 mice as previously described (Duong *et al.*, 1999; Fischer *et al.*, 2011; Fratta *et al.*, 2012) Ventral horn spinal cords from embryonic Day 13 mice were isolated and dissociated with 0.025% trypsin and motor neurons were purified using OptiPrep™ density gradient centrifugation. Motor neurons were resuspended in Neurobasal® medium and grown on polyornithine and laminin coated plates for 7 days. Motor neuron cultures were treated with 50 nM dihydrotestosterone for 3 days before use.

In some experiments, the sex of the embryos was established by PCR amplification of the *SrY* gene found exclusively on the Y chromosome, using myogenin (present on both the X and Y chromosomes) as a control, according to the method described by McClive and Sinclair (2001).

### Measurement of cytosolic and endoplasmic reticulum calcium ions

Endoplasmic reticulum Ca^2+^ concentration can be indirectly determined in cells *in vitro,* by measuring the changes in cytosolic Ca^2+^ levels that occur following application of drugs that deplete endoplasmic reticulum Ca^2+^. However, to achieve this, the cells must be in a Ca^2+^-free medium to ensure that the endoplasmic reticulum is the only source of any observed change in cytosolic Ca^2+^ levels.

Cytosolic Ca^2+^ levels were examined at 7 days *in vitro*, in untreated and dihydrotestosterone-treated (50 nM) wild-type and AR100 motor neurons, using confocal microscopy in combination with drugs that alter cellular Ca^2+^ handling as previously described (Bassik *et al.*, 2004; Bilsland *et al.*, 2008; Bravo *et al.*, 2011). Confocal images were obtained using a Zeiss 510 confocal laser scanning microscope using a ×40 oil-objective lens. Cells were kept at 37°C using a heated stage. Coverslips (22 mm) were loaded with 500 µl of 5 µM Fluo-4 AM (Molecular Probes, Invitrogen) and 0.005% pluronic acid diluted in confocal recording medium and incubated in the dark for 30 min at 37°C. Fluo-4 AM, a non-ratiometric or single wave dye that measures free cytosolic calcium, was excited at 488 nm and the emission fluorescence was measured at 518 nm. A time-lapse image was taken once every second for 800 s and background fluorescence was subtracted from the selected motor neurons. Motor neurons were identified using specific morphological criteria: a cell body diameter of >15 µm and possessing more than two neuritic processes, as previously described (Bilsland *et al.*, 2008). The fluorescence values that were obtained were then calibrated into a Ca^2+^ concentration in nanometres using the following equation (Maravall *et al.*, 2000):

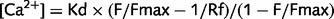

Where the values Kd = 350 nM; Fmax = maximum fluorescence point following ionomycin application; and Rf = 100 were used.

Some experiments were validated using Fura-2, a ratiometric dye, to take into account some of the disadvantages of using a single wave dye such as Fluo-4 AM. In these experiments, the ORCA imaging system (Hamamatsu) was used (Abramov and Duchen, 2008). Coverslips were incubated with 500 µl of 5 µM Fura-2 (Molecular probes, Invitrogen) and 0.005% pluronic acid diluted in confocal recording medium. Again, coverslips were incubated for 30 min at 37°C and 5% CO_2_ in the dark. Fura-2 was excited at 340 nm and 380 nm and the emission fluorescence was measured at 505 nm. A ratio between the two maxima at the different wavelengths was calculated by dividing the 340 nm fluorescence value at a given time point by the 380 nm fluorescence value after background subtraction. Time-lapse images were taken at 4-s intervals.

**Figure 2:**
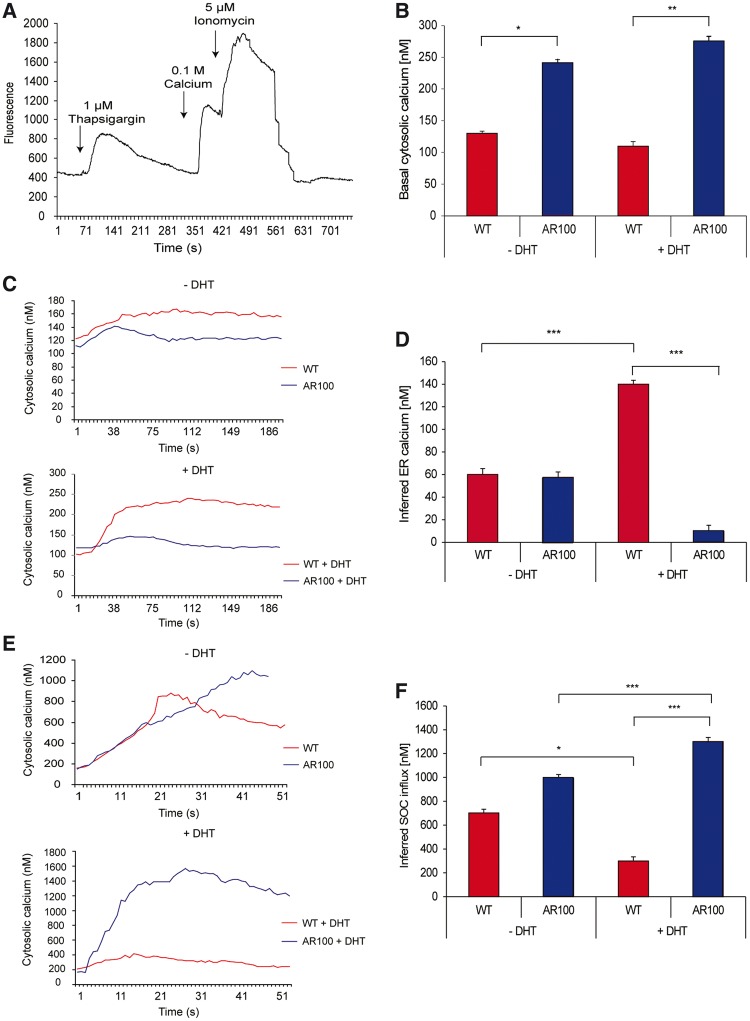
Endoplasmic reticulum Ca^2+^ homeostasis is dysregulated in cultured motor neurons from SBMA muscular atrophy mice. Primary embryonic motor neurons from wild-type (WT) and AR100 SBMA mice were imaged after 7 days in culture to monitor cytosolic Ca^2+^ levels. (**A**) The trace illustrates the protocol used to measure basal cytosolic Ca^2+^ and infer endoplasmic reticulum Ca^2+^ and store-operated Ca^2+^ influx and is described in the main text. (**B**) Basal cytosolic Ca^2+^ levels were measured in primary AR100 and wild-type motor neurons in a recording medium containing 1 mM Ca^2+^. Significantly higher [Ca^2+^] was measured in AR100 compared to wild-type motor neurons both in the absence and presence of 50 nM dihydrotestosterone (DHT) for 72 h (*n* ≥ 31, *P* = 0.02 and 0.002 respectively, ANOVA with Bonferroni post hoc analysis; *n* = number of motor neurons from at least three replicate cultures). (**C**) Cytosolic Ca^2+^ responses to thapsigargin exposure in wild-type and AR100 motor neurons were established in the absence and presence of dihydrotestosterone. (**D**) The increase in cytosolic Ca^2+^ levels in AR100 and wild-type motor neurons following thapsigargin application in a Ca^2+^ free recording medium was used to infer levels of endoplasmic reticulum Ca^2+^. A significantly lower level of inferred endoplasmic reticulum Ca^2+^ was present in AR100 motor neurons compared to wild-type controls in the presence of dihydrotestosterone (*n* ≥ 31, *P* = 0.002, ANOVA with Bonferroni post hoc analysis). (**E**) Following addition of Ca^2+^ in the recording media, cytosolic [Ca^2+^] was measured in wild-type and AR100 motor neurons in the absence and presence of dihydrotestosterone treatment. (**F**) The increase in cytosolic Ca^2+^ levels in primary AR100 and wild-type motor neurons after external Ca^2+^ introduction was measured and was used to infer levels of store-operated Ca^2+^ influx. Significantly higher levels of inferred store-operated Ca^2+^ influx in AR100 motor neurons compared to wild-type in the presence of dihydrotestosteronewere observed (*n* ≥ 31, *P* = 0.027, ANOVA with Bonferroni post hoc analysis). Error bars show the SEM. *P < 0.05, **P < 0.01, ***P < 0.001.

Immediately before the start of the protocol, the culture medium containing Ca^2+^ was replaced with a Ca^2+^-free imaging medium (125 mM NaCl, 5 mM KCl, 1 mM Na_3_PO_4_.12H_2_O, 1 mM MgSO_4_.7H_2_O, 1g/l d-Glucose, 20 mM HEPES) and a basal level of cytosolic fluorescence was obtained. To measure endoplasmic reticulum Ca^2+^ content, ∼45 s later, the cells were treated with 1 µM thapsigargin, a non-competitive inhibitor of the SERCA pump in the endoplasmic reticulum membrane (Michelangeli and East, 2011), which depletes the endoplasmic reticulum of Ca^2+^. Therefore, any increase in cytosolic Ca^2+^ will be a reflection of endoplasmic reticulum Ca^2+^ as there is no external Ca^2+^. The effects of thapsigargin are complete within 300 s and so the cells were left undisturbed for this time. After endoplasmic reticulum Ca^2+^ depletion, Ca^2+^ was reintroduced into the external medium, allowing store-operated Ca^2+^ influx to occur in response to the depletion of endoplasmic reticulum Ca^2+^, which resulted in an increase in cytosolic Ca^2+^. To calibrate the fluorescence measurements, the maximum cytosolic Ca^2+^ levels (fluorescence) that could be obtained were determined 60 s later by treating the cells with the Ca^2+^ ionophore, ionomycin (5 µM), in the presence of extracellular Ca^2+^, thereby achieving a maximum level of cytosolic Ca^2+^; this permits calibration of fluorescence into a Ca^2+^ concentration in nM (Fig. 2A).

In experiments using Fluo-4 AM, cytosolic Ca^2+^ was analysed using LSM software (Carl Zeiss in association with EMBL). In experiments using Fura-2, the concentration was analysed using AQM software (Tech Direct). In all experiments background fluorescence was deducted from fluorescence measurements before calibration.

### Tissue collection

Spinal cord tissue was collected from wild-type and AR100 mice at different ages: 5 days postnatal and 3, 12 and 18 months. The mice were terminally anaesthetized with phenobarbitone and were transcardially perfused with 0.9% saline. For western blot analysis, whole spinal cords were dissected, snap frozen in liquid nitrogen and stored at −80°C until processed. For immunohistochemical analysis, after perfusion with saline, the mice were fixed with 4% paraformaldehyde, the lumbar spinal cords (L2–L6) removed, post fixed in 4% paraformaldehyde and cryoprotected in 30% sucrose at 4°C overnight. Serial transverse sections were then cut on a cryostat at 10 µm and stored at −80°C until processed. The tissues and cells were then examined for the expression of a variety of markers by western blot and immunostaining, using the antibodies summarized in Supplementary Table 1.

### Western blot analysis

Protein levels of a number of markers (Supplementary Table 1) were quantified in cells in culture at 7 days *in vitro* and in spinal cord homogenates using western blot as described previously (Malik *et al.*, 2008, 2012).

Briefly, protein samples were separated by SDS-PAGE and transferred onto a nitrocellulose membrane (Tocris). Samples were then incubated with primary antibodies (Supplementary Table 1) overnight at 4°C followed by horseradish peroxidase conjugated secondary antibodies (Dako). The blots were imaged and the density was analysed using FluorChem®SP imaging software (Cell Biosciences).

### Immunofluorescence and immunohistochemistry

Motor neuron cultures and spinal cord sections were immunostained for a variety of markers using the antibodies shown in Supplementary Table 1. For cells in culture, the cells were fixed at 7 days *in vitro* in 4% paraformaldehyde for 15–20 min at room temperature, and then washed three times for 5 min in PBS.

For immunostaining, spinal cord sections and coverslips were incubated at room temperature for 1 h in blocking solution, which consisted of 5% milk and 3% serum (source dependant on secondary antibody) in PBS-0.1% Triton™ X-100 (Sigma). The slides were incubated at 4°C overnight in the primary antibody (Supplementary Table 1), followed by an appropriate secondary antibody. In the case of biotinylated secondary antibodies, (but not fluorescently-conjugated antibodies), the slides were incubated in an avidin tertiary antibody (diluted in PBS) and the cells then stained for the nuclear marker, DAPI (4’6 diamidino-2-phenylindole; 1:2000 in PBS). A negative control, in which the primary antibody was omitted, was also included.

### Statistical analysis

Statistical analysis was performed using Microsoft Excel and SPSS v15. To test for significance, non-parametric data analysis was performed using the Kruskal-Wallis test and for parametric data, either a two sample t-test or a one-way ANOVA with a Bonferoni *post hoc* test was carried out for pairwise comparison between all groups within the experiment. A *P*-value of <0.05 was considered statistically significant.

## Results

### Dysregulation of endoplasmic reticulum calcium homeostasis in SBMA motor neurons *in vitro*

Endoplasmic reticulum stress has been reported to be elevated in several neurodegenerative disorders including models of polyglutamine repeat expansion (Reijonen *et al.*, 2008) and motor neuron disease (Saxena *et al.*, 2009), and may be dependent on dysregulation of Ca^2+^ handling (Ma *et al.*, 2002). To examine the role of endoplasmic reticulum stress in SBMA, we first measured the levels of Ca^2+^ within the cytoplasm and endoplasmic reticulum of cultured embryonic motor neurons derived from wild-type and AR100 SBMA mice. Measurements were performed in the presence or absence of dihydrotestosterone, the androgen receptor ligand, and the presence or absence of Ca^2+^ in the external recording medium. Treatment with dihydrotestosterone results in translocation of the androgen receptor into the nucleus and was therefore used to model the ligand (androgen)-dependant characteristics of the disease (Takeyama *et al.*, 2002; Walcott and Merry, 2002). Both the time course and the magnitude of the Ca^2+^ response were examined.

Basal cytosolic Ca^2+^ levels were first determined in wild-type and AR100 motor neurons in dihydrotestosterone-treated and untreated cultures in the presence of external Ca^2+^ (1 mM). As shown in Fig. 2B, treatment with dihydrotestosterone had no effect on cytosolic Ca^2+^ levels in wild-type motor neurons. However, there was a significant difference in basal cytosolic Ca^2+^ levels between untreated wild-type (130 ± 3.71 nM, *n* = 32) and untreated AR100 motor neurons (240 ± 6.43 nM, *n* = 28, *P* = 0.02, ANOVA) in the presence of external Ca^2+^. Furthermore, cytosolic Ca^2+^ levels were found to be significantly elevated in dihydrotestosterone-treated AR100 motor neurons (275 ± 7.60 nM, *n* = 53) compared to dihydrotestosterone-treated wild-type motor neurons (110 ± 7.18 nM, *n* = 31, *P* = 0.002, ANOVA). In a Ca^2+^-free recording medium, the differences in cytosolic Ca^2+^ levels between dihydrotestosterone-treated and untreated wild-type and AR100 motor neurons were almost completely eliminated, indicating that they primarily represent increased Ca^2+^ influx in AR100 and dihydrotestosterone-treated AR100 cells under basal conditions (Supplementary Fig. 1).

We next examined the effects of mutant androgen receptor activation on the levels of inferred endoplasmic reticulum Ca^2+^ levels, determined by measuring cytosolic Ca^2+^ levels following application of thapsigargin, which depletes the endoplasmic reticulum of Ca^2+^. Examination of the thapsigargin-induced changes in cytosolic Ca^2+^ revealed a difference in the response kinetics between wild-type and AR100 cultured motor neurons, irrespective of dihydrotestosterone treatment (Fig. 2C). Following exposure to thapsigargin, the cytosolic Ca^2+^ response had similar onset latencies in wild-type and AR100 motor neurons, with the response almost instantaneous, reaching a peak within 40 to 50 s. However, the response seemed much more transient in AR100 motor neurons, in which Ca^2+^ levels fell by 10 nm after reaching maximum, in contrast to wild-type motor neurons where Ca^2+^ levels remained steady after reaching maximum, at least for the duration of the recording. This pattern of response kinetics was the same in cultures either treated or left untreated with dihydrotestosterone.

However, there was a significant difference in the magnitude of the thapsigargin-induced change in cytosolic Ca^2+^ (i.e. inferred endoplasmic reticulum Ca^2+^) in wild-type and AR100 motor neurons treated with dihydrotestosterone (Fig. 2C and D). In the absence of dihydrotestosterone treatment, there was no difference in inferred endoplasmic reticulum Ca^2+^ levels between wild-type (60 ± 5.30 nM, *n* = 32) and AR100 motor neurons (57.5 ± 4.82 nM, *n* = 28; Fig. 2D). Treatment with dihydrotestosterone resulted in a clear increase in inferred endoplasmic reticulum Ca^2+^ levels in wild-type motor neurons (140 ± 3.41 nM, *n* = 31) compared to untreated cultures (*P* < 0.005, ANOVA). In contrast, in dihydrotestosterone-treated AR100 motor neurons, inferred endoplasmic reticulum Ca^2+^ levels were significantly lower (10 ± 5.07 nM, *n* = 35) than in treated wild-type motor neurons (*P* < 0.005, ANOVA; Fig. 2D), indicating that dihydrotestosterone treatment in AR100 motor neurons leads to depletion of the endoplasmic reticulum Ca^2+^ store, a hallmark of the endoplasmic reticulum stress response (Chami *et al.*, 2008).

To further investigate the effects of the AR100 expansion on cellular Ca^2+^ handling, Ca^2+^ was reintroduced into the external medium, allowing store-operated Ca^2+^ influx to occur. Store-operated Ca^2+^ influx was determined by measuring the increase in cytosolic Ca^2+^ following treatment with thapsigargin in the presence of external Ca^2+^ (Fig. 2E and F). In non-dihydrotestosterone treated motor neurons, maximum cytosolic Ca^2+^ levels were reached more rapidly in wild-type motor neurons than in AR100 motor neurons (Fig. 2E). However, the elevation in cytosolic Ca^2+^ was more prolonged in both dihydrotestosterone-treated and untreated AR100 motor neurons than wild-type. The magnitude of store-operated Ca^2+^ influx also differed between dihydrotestosterone-treated and untreated cultures, as well as in wild-type and AR100 motor neurons (Fig. 2F). In wild-type cultures, inferred store-operated Ca^2+^ influx was reduced in dihydrotestosterone-treated motor neurons compared to untreated motor neurons (*P* = 0.05, ANOVA). Thus, in dihydrotestosterone-treated wild-type motor neurons, cytosolic Ca^2+^ levels were ∼400 nM lower than in untreated motor neurons. In contrast, in AR100 cultures, dihydrotestosterone treatment increased inferred store-operated Ca^2+^ influx compared to untreated cells. Thus in dihydrotestosterone-treated AR100 motor neurons, following application of thapsigargin, cytosolic Ca^2+^ levels rose to 1300 ± 34.63 nM (*n* = 35; *P* < 0.05, ANOVA) compared with 950 nM in untreated AR100 motor neurons. Furthermore, store-operated Ca^2+^ influx was also significantly higher in dihydrotestosterone-treated AR100 motor neurons than in dihydrotestosterone-treated wild-type motor neurons (300 ± 34.11 nM, *n* = 31; *P* < 0.027, ANOVA; Fig. 2E). These findings suggest that the activation of the polyglutamine-expanded androgen receptor by dihydrotestosterone, in parallel with endoplasmic reticulum Ca^2+^ store depletion, activates store-operated Ca^2+^ influx, leading to higher cellular Ca^2+^ load of cultured AR100 motor neurons (Fig. 2F).

As Fluo-4 AM, a single wave dye, was used to determine cytosolic Ca^2+^ levels in the experiments described above, cellular Ca^2+^ handling in AR100 and wild-type motor neurons was also examined using the ratiometric dye, Fura-2. These dyes have different sensitivities and Ca^2+^ associates and dissociates at different rates from each of the dyes. As shown in Supplementary Fig. 2, the pattern of cellular Ca^2+^ handling observed with Fluo-4 AM was identical when experiments were repeated using Fura-2, which demonstrated that the findings summarized in Fig. 2 are not specific to a particular Ca^2+^ indicator.

In addition, as SBMA is a ligand-dependent disease that only manifests in males due to the activation of the androgen receptor by androgens, we also examined Ca^2+^ handling in motor neurons from male and female wild-type and AR100 mice and found no significant difference (Supplementary Fig 3). Therefore, the differences in Ca^2+^ handling between wild-type and AR100 motor neurons described above were dependent on availability of ligand and activation of the androgen receptor. Taken together, these findings show that in cultured motor neurons of SBMA mice, the presence of dihydrotestosterone causes a depletion of endoplasmic reticulum Ca^2+^ and activation of store-operated Ca^2+^, resulting in an abnormal Ca^2+^ load in the cytosol, reflected by increased basal cytosolic Ca^2+^. These effects are as a result of ligand-dependant activation of the mutant expanded androgen receptor.

### Loss of the endoplasmic reticulum calcium pump and activation of the endoplasmic reticulum stress response in SBMA motor neurons *in vitro*

**Figure 3:**
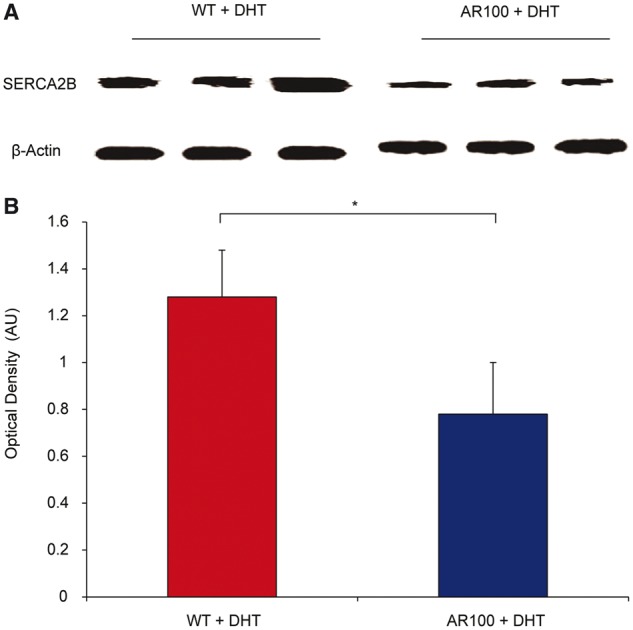
The expression of SERCA2b, an endoplasmic reticulum calcium transport pump protein, is reduced in cultured motor neurons from SBMA. (**A**) SERCA pump (SERCA2b) is responsible for replenishing endoplasmic reticulum Ca^2+^ and the levels of the protein were quantified using western blot, using samples from at least three separate wild-type (WT) and AR100 purified cultured primary motor neuron cultures subjected to a 72 h 50 nM dihydrotestosterone (DHT) treatment. Antibodies against SERCA2b, and the loading control, actin. (**B**) The expression level of SERCA2b was quantified using densitometry and was 40% lower in AR100 primary motor neurons than in wild-type controls (*n* = 3, *P* = 0.04, *t*-test, *n* = number of replicate cultures). Error bars show the SEM. * *P* < 0.05. AU = arbitrary units.

Expression of the SERCA pump (SERCA2b isoform) has previously been shown to be regulated downstream of androgen receptor activation (Foradori and Handa, 2008). We therefore examined the expression of the SERCA2b pump protein to establish whether altered expression of SERCA2b may explain the abnormal Ca^2+^ handling observed in AR100 motor neurons in the presence of dihydrotestosterone (Fig. 3). Using purified cultures of primary wild-type and AR100 motor neurons, we found that the levels of SERCA2b were significantly decreased in dihydrotestosterone-treated AR100 motor neurons by nearly 40% (0.78 ± 0.22, *n* = 6, *P* = 0.04, *t*-test) compared to wild-type (1.28 ± 0.2, *n* = 6; Fig. 3). Therefore, the disruption of Ca^2+^ homeostasis observed in AR100 motor neurons and summarized in Fig. 2 may, at least in part, be explained by a decrease in the expression of the SERCA2b endoplasmic reticulum calcium pump.

**Figure 4:**
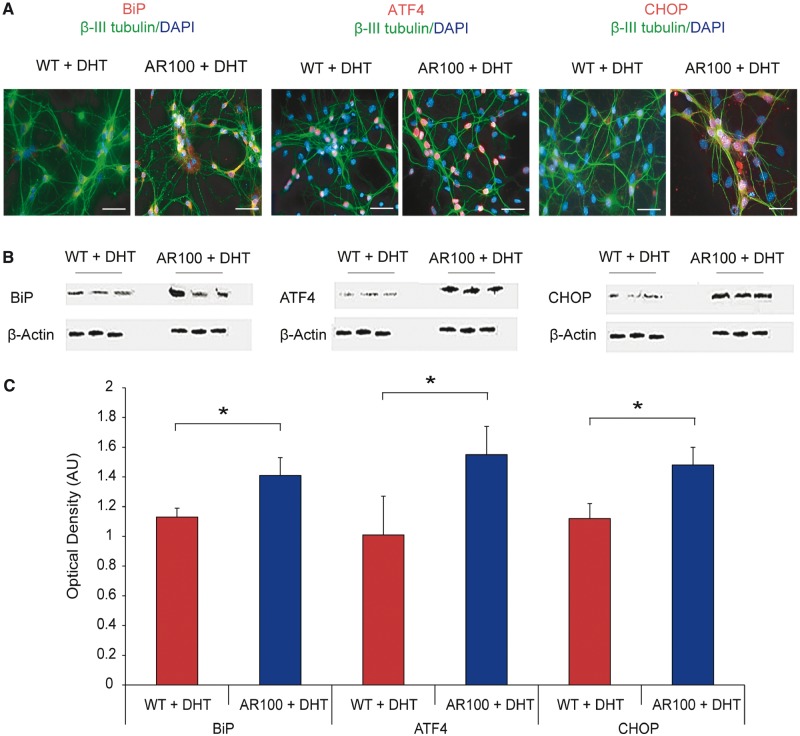
Markers of endoplasmic reticulum stress are increased in cultured motor neurons from SBMA mice. (**A**) The PERK-activated pathway of the unfolded protein response was examined to establish the role of endoplasmic reticulum stress in motor neurons. Cultures were immunostained for a neuronal marker (β-III tubulin, green), a nuclear marker (DAPI, blue) and endoplasmic reticulum stress markers BiP, ATF4 or CHOP (all red). In all cases immunoreactivity for the endoplasmic reticulum stress markers appeared to be elevated in AR100 motor neurons compared to wild-type (WT) controls. Scale bars in images are 30 µm. (**B**) Purified primary motor neuron cultures from at least three different wild-type and AR100 embryos were analysed by western blot to quantify the levels of BiP, ATF4 and CHOP proteins. (**C**) Densitometry showed that the expression levels of BiP, ATF4 and CHOP were significantly higher in purified AR100 motor neurons than in wild-type controls (*n* = 3, *P* = 0.05, 0.047 and 0.008 respectively, *t*-test, *n* = number of mice). Error bars show the SEM. *P < 0.05. AU = arbitrary units.

Because alterations in Ca^2+^ homeostasis can lead to an increased susceptibility to endoplasmic reticulum stress, we examined cultures of wild-type and AR100 dihydrotestosterone-treated embryonic motor neurons for markers of endoplasmic reticulum stress including BiP, ATF4 and CCAAT-enhancer-binding protein homologous protein (CHOP) to investigate the PERK-activated branch of the unfolded protein response (Fig. 1). We observed an increase in the expression of all the markers examined in dihydrotestosterone-treated AR100 motor neurons compared to treated wild-type controls (Fig. 4A). In the case of BiP, the expression was predominantly found in the cytosol of motor neurons, whereas ATF4 and CHOP were almost exclusively nuclear. The expression of endoplasmic reticulum stress markers was estimated using western blot analysis, and the results showed that compared to wild-type motor neurons, there was an increase in the expression of BiP, ATF4 and CHOP in AR100 motor neurons (*P* < 0.05, *t*-test, for all markers, wild-type versus AR100). These results show that markers of endoplasmic reticulum stress are noticeably increased in ligand-treated embryonic motor neurons of SBMA mice, indicative of an endoplasmic reticulum stress response.

### Markers of endoplasmic reticulum stress are increased in spinal cords of SBMA mice before symptom onset

**Figure 5:**
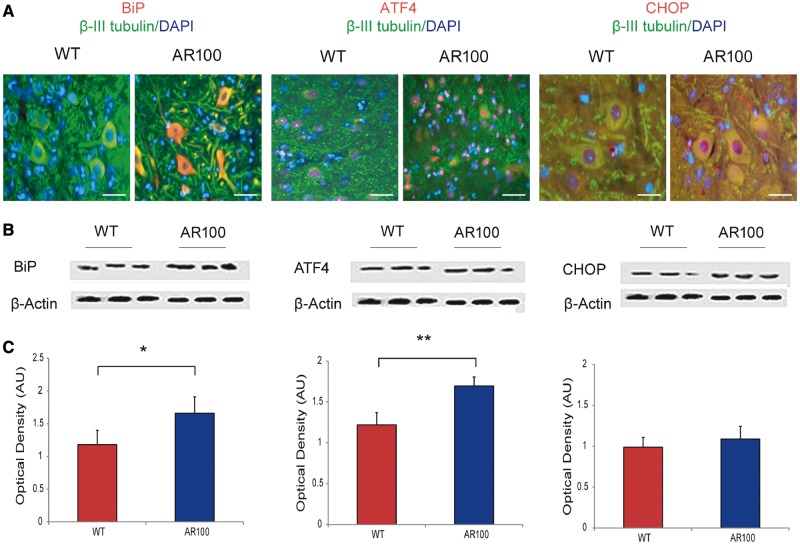
Endoplasmic reticulum stress markers are elevated in the spinal cord of SBMA mice before the onset of symptoms. (**A**) Spinal cord lumbar 10 µM sections from AR100 and wild-type (WT) littermates at 3 months of age were then immunostained for either BiP, ATF4 or CHOP (all red) and co-stained for β-III tubulin (green), a neuronal marker and DAPI (blue), a nuclear marker. An increase in BiP and ATF4 immunoreactivity was observed in the cytoplasm of AR100 motor neurons. A modest increase in CHOP immunoreactivity was also detected. Scale bars in **A** = 40 µm. (**B**) Spinal cords from at least three different wild-type and AR100 mice at 3 months of age were analysed by western blot to quantify the levels of BiP, ATF4 and CHOP proteins. (**C**) Quantification by densitometry shows expression of BiP was significantly higher level in AR100 spinal cord compared to wild-type controls (*n* = 3, *P* = 0.05, *t*-test, *n* = number of mice). ATF4, was also increased in AR100 spinal cord compared to wild-type controls (*n* = 3, *P* = 0.002, *t*-test) but there was no significant difference in expression at 3 months of age of CHOP. Error bars show the SEM. *P < 0.05, **P < 0.01. AU = arbitrary units.

AR100 mice develop a late-onset progressive neuromuscular phenotype from ∼12 months of age (Sopher *et al.*, 2004; Malik *et al.*, 2011, 2013). As AR100 embryonic motor neurons expressed elevated levels of markers of endoplasmic reticulum stress, we examined whether these markers were also elevated *in vivo* in spinal cords of AR100 mice at various stages of disease. Figure 5A shows the typical pattern of expression of BiP, ATF4 and CHOP in spinal cord ventral horns of wild-type and AR100 mice at 3 months of age. The expression of these markers was also examined by western blot (Fig. 5B), and quantified (Fig. 5C) not only in spinal cords of 3-month-old mice, but also mice aged 5 days, and 12 and 18 months (Supplementary Fig. 4). We noted an increase in the expression of BiP, ATF4 and CHOP in spinal cords of AR100 mice, although this increase did not reach significance for all markers at all the different ages, particularly at late stage of the disease at 18 months of age. However, there was a clear increase in the expression of all endoplasmic reticulum stress markers in the spinal cord of presymptomatic AR100 mice at 5 days of age, well before the onset of any disease symptoms. Expression of these markers was not found to be elevated in mice at 18 months of age likely reflecting the extensive motor neuron loss that is known to occur by this late stage of disease in AR100 mice (Malik *et al.*, 2013; Supplementary Fig. 4).

**Figure 6:**
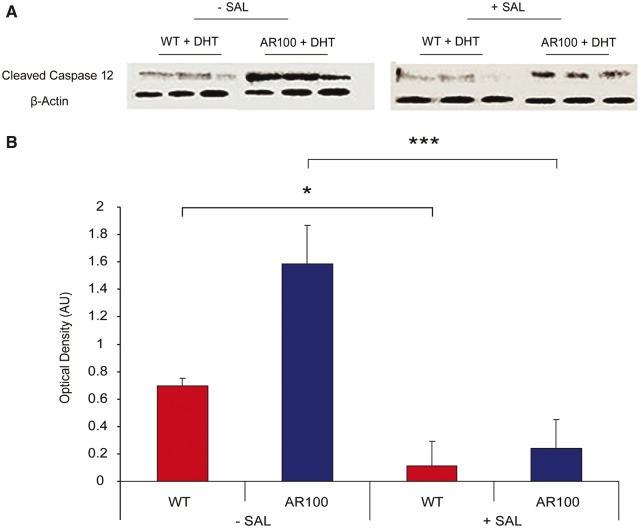
Treatment of cultured motor neurons with an endoplasmic reticulum stress inhibitor reduces endoplasmic reticulum stress-induced apoptosis. Purified embryonic primary wild-type (WT) and AR100 motor neuron were cultured for 7 days and were treated for 72 h with 50 nM dihydrotestosterone (DHT). Cultures were analysed by western blot to examine the levels of cleaved active caspase 12 in the absence and presence of 24 h salubrinal treatment. In the absence of salubrinal treatment there was a significantly higher level of active caspase 12 in AR100 motor neurons compared to the wild-type (*n* = 3, *P* = 0.003, *t*-test, *n* = number of replicate cultures). Following 24 h of salubrinal treatment, however, there was a significant decrease in caspase 12 expression in both wild-type and AR100 motor neurons (*n* = 3, *P* = 0.042 and 0.0004 respectively, *t*-test) and no longer a significant difference in expression between wild-type and AR100 motor neurons. Error bars show the SEM. *P < 0.05, **P < 0.01, ***P < 0.001. AU: arbitrary units.

To establish whether this reduction in motor neuron survival was related to the endoplasmic reticulum stress detected in AR100 motor neurons (Figs 4 and 5), cultures were treated with salubrinal, a selective inhibitor of EIF2A dephosphorylation, and consequently endoplasmic reticulum stress-mediated apoptosis (Kessel, 2006). A dose-response profile of salubrinal was first established in wild-type motor neurons in order to identify any toxic effects (data not shown). Motor neuron cultures were subsequently treated at 7 days *in vitro* with 500 nM salubrinal for 24 h, a dose at which salubrinal was not detrimental to motor neuron survival, but previously shown to be effective in inhibiting endoplasmic reticulum stress *in vitro* (Kessel, 2006). Treatment with salubrinal improved the survival of both wild-type and AR100 motor neurons, although the effect was more pronounced in AR100 cultures (Fig. 6A). Following treatment with salubrinal, more wild-type motor neurons survived than in untreated wild-type cultures (*P* < 0.01, *t*-test). In AR100 cultures however, treatment with salubrinal resulted in an even greater improvement in motor neuron survival, and more AR100 motor neurons survived than in untreated AR100 cultures (*P* < 0.001, *t*-test, Fig. 6A). Salubrinal may therefore prevent endoplasmic reticulum stress-mediated apoptosis and improve AR100 motor neuron survival. These findings show that endoplasmic reticulum stress is activated in spinal cord motor neurons of SBMA mice and that this occurs well before symptom onset, suggesting that endoplasmic reticulum stress may play a causal role in the development and pathology of this disease.

### Endoplasmic reticulum stress-mediated apoptosis is activated in SBMA motor neurons *in vitro* and is reduced by treatment with salubrinal

To examine whether the endoplasmic reticulum stress detected in AR100 motor neurons led to the activation of the endoplasmic reticulum-associated cell death pathway, we examined the protein levels of cleaved caspase 12 in purified embryonic primary motor neuron cultures, treated with dihydrotestosterone, by western blot (Fig. 6A and B). Endoplasmic reticulum stress induced apoptosis is mediated by caspase 12, which is primarily located within the endoplasmic reticulum. The expression of activated caspase 12 was found to be significantly higher in AR100 motor neurons (*P* = 0.003, *t*-test) than in wild-type controls. Treatment of dihydrotestosterone-treated purified embryonic motor neurons with salubrinal resulted in a significant decrease in caspase 12 expression in both wild-type and AR100 motor neurons, although the decrease was much more dramatic in AR100 motor neurons (Fig. 6A and B). Therefore, treatment with an endoplasmic reticulum stress inhibitor prevented the activation of the endoplasmic reticulum-associated cell death pathway in AR100 motor neurons.

## Discussion

The results presented in this study show that dysregulation of Ca^2+^ homeostasis in motor neurons of SBMA AR100 mice resulted in endoplasmic reticulum stress**,** which in turn increased the vulnerability of AR100 motor neurons to activation of endoplasmic reticulum-stress-induced apoptosis. Endoplasmic reticulum stress was reduced by treatment with salubrinal and was found not only to be a feature of AR100 motor neurons in culture, but also *in vivo*, where an increase in the expression of stress markers in spinal motor neurons of AR100 mice as young as 5 days of age was observed. Taken together, these results suggest that endoplasmic reticulum stress is an early pathological feature of SBMA, and may play a causal, rather than secondary role in the pathogenesis of this disease.

Endoplasmic reticulum stress has been proposed to play an important role in the pathogenesis of the motor neuron disorder amyotrophic lateral sclerosis as well as in polyglutamine repeat disorders, such as Huntington’s disease (Reijonen *et al.*, 2008; Saxena *et al.*, 2009). In amyotrophic lateral sclerosis, endoplasmic reticulum stress markers including BiP and CHOP are elevated in motor neurons of *SOD1^G93A^* mice that model the disease (Saxena *et al.*, 2009), as well as in spinal cord autopsy tissue of patients with the disease (Atkin *et al.*, 2008). Furthermore, inhibition of endoplasmic reticulum stress with salubrinal significantly improved motor neuron survival both *in vitro* and in *SOD1^G93A^* mice *in vivo* (Saxena *et al.*, 2009). Likewise, transfection of cultured cells with a polyglutamine-expanded huntingtin protein increased the expression levels of BiP and CHOP, and inhibition of endoplasmic reticulum stress with salubrinal improved cell survival (Reijonen *et al.*, 2008). Furthermore, independent studies have shown that the mutant polyglutamine-expanded androgen receptor, responsible for SBMA, activates the unfolded protein response, an endoplasmic reticulum protein quality control pathway (Szegezdi *et al.*, 2006), both *in vitro* (Thomas *et al.*, 2005) and *in vivo*, in a mouse model of SBMA (Yu *et al.*, 2011). However, despite the evidence for a causal role of endoplasmic reticulum stress in the pathogenesis of motor neuron and polyglutamine repeat diseases, its role in SBMA, which is both a motor neuron and polyglutamine disorder, has yet to be fully established.

The results of this study show that disruption of Ca^2+^ handling occurs in cultured embryonic motor neurons from SBMA mice. We observed a higher concentration of cytosolic Ca^2+^ in SBMA AR100 motor neurons treated with the androgen receptor ligand dihydrotestosterone, than in dihydrotestosterone-treated wild-type motor neurons. This finding suggests that AR100 motor neurons may potentially be more vulnerable to excitotoxic insults that elevate cytosolic Ca^2+^ even further above normal in SBMA motor neurons. A similar finding has been reported to occur in other models of motor neuron disease (Appel *et al.*, 2001). Elevated levels of cytosolic Ca^2+^ are associated with several harmful processes including free radical production, lipid peroxidation and activation of apoptosis (Kakkar *et al.*, 1992; Demaurex and Distelhorst, 2003). Thus, any underlying pathology that elevates cytosolic Ca^2+^ levels above normal will render cells more vulnerable to stimuli that can further increase Ca^2+^ levels. The inferred endoplasmic reticulum Ca^2+^ concentration observed in AR100 motor neurons after application of thapsigargin was lower in dihydrotestosterone-treated AR100 motor neurons than in dihydrotestosterone-treated wild-type motor neurons. Therefore, endoplasmic reticulum stress is more likely to occur in AR100 motor neurons, as the normal function of the endoplasmic reticulum is dependent on Ca^2+^ (Paschen and Doutheil, 1999). Furthermore, exposure to dihydrotestosterone had opposite effects in wild-type and AR100 motor neurons: it increased endoplasmic reticulum Ca^2+^ in wild-type motor neurons whereas it significantly depleted it in AR100 motor neurons, which may be detrimental to cell survival (Demaurex and Distelhorst, 2003). The effect of dihydrotestosterone treatment on endoplasmic reticulum Ca^2+^ may reflect the ligand dependant pathophysiology of SBMA and reproduces the gender specificity of the disease, i.e. severe symptoms usually only manifest in males as they have higher levels of the androgen receptor ligand, testosterone, than females (Katsuno *et al.*, 2002, 2012). Furthermore, in accordance with the reduced levels of endoplasmic reticulum Ca^2+^, store-operated Ca^2+^ influx was elevated in dihydrotestosterone-treated AR100 motor neurons to compensate for the endoplasmic reticulum Ca^2+^ deficit (Smani *et al.*, 2004). Additionally, the kinetics of Ca^2+^ homeostasis was also altered in AR100 motor neurons so that after treatment with thapsigargin, the resulting change in cytosolic Ca^2+^ in AR100 motor neurons was more transient than in wild-type controls. This may indicate that Ca^2+^ expulsion from the cytosol is overactive in AR100 motor neurons. However, since the increase in cytosolic Ca^2+^ associated with inferred store-operated Ca^2+^ influx was more prolonged in AR100 motor neurons than in wild-type controls, it is more likely that the transient nature of the Ca^2+^ response to thapsigargin in AR100 motor neurons was the result of a lower endoplasmic reticulum Ca^2+^, so that Ca^2+^ is depleted more rapidly, and as a consequence, the response is shorter.

One of the downstream effects of androgen receptor activation is transcription of the SERCA pump, which provides a route by which Ca^2+^ enters the endoplasmic reticulum lumen (Foradori and Handa, 2008). The SERCA pump may therefore provide a link between the activation of the androgen receptor and offer an explanation for the observed depletion in endoplasmic reticulum Ca^2+^. We found that SERCA2b expression was significantly lower in AR100 motor neurons than in wild-type controls, which could reduce sequestration of Ca^2+^ from the cytosol into the endoplasmic reticulum lumen, giving rise to increased cytosolic Ca^2+^ and lower endoplasmic reticulum Ca^2+^. In conjunction with elevated cytosolic Ca^2+^, the unfolded protein response markers BiP, ATF4 and CHOP were all significantly elevated in dihydrotestosterone-treated AR100 motor neurons compared with wild-type controls. AR100 motor neurons can therefore be considered to have a higher basal level of endoplasmic reticulum stress. Although loss of SERCA2b expression may in part be due to diminution of transcriptional activity of androgen receptor, our data suggest that generation of endoplasmic reticulum stress is by activation of the mutant expanded androgen receptor. In cultured motor neurons of AR100 SBMA mice, dihydrotestosterone treatment enhances the dysregulation of endoplasmic reticulum Ca^2+^ homeostasis compared with untreated cells and strongly suggests the pathological effects and generation of endoplasmic reticulum stress are related to the ligand-dependant activation of the mutant expanded androgen receptor.

To determine whether endoplasmic reticulum stress-induced apoptosis contributes to the vulnerability of AR100 motor neurons, the expression of activated caspase 12 was examined. We found that the levels of activated caspase 12, which is located primarily within the endoplasmic reticulum, was significantly higher in AR100 motor neurons than in wild-type controls, indicating increased activation of the endoplasmic reticulum-associated cell death pathway. We next examined the effects of the endoplasmic reticulum stress inhibitor salubrinal. We found that salubrinal significantly reduced the level of caspase 12 expression in AR100 motor neurons, suggesting that inhibition of endoplasmic reticulum stress may prevent endoplasmic reticulum stress-induced apoptosis by suppressing the activation of caspase 12. Although there is strong evidence to suggest that salubrinal predominantly inhibits endoplasmic reticulum stress-induced apoptosis through inhibition of dephosphorylation of EIF2A (Boyce *et al.*, 2005), other effects have been noted. The inhibition of dephosphorylation of EIF2A has also been linked to the inhibition of translocation of p53 to the mitochondria, thus counteracting mitochondrial-associated apoptosis (Lee and Kim, 2013). Therefore, the effects of salubrinal that are independent of endoplasmic reticulum stress could also possibly play a role. However, due to the decrease in activation of caspase 12 in SBMA motor neurons after salubrinal treatment, the prevention of endoplasmic reticulum stress-induced apoptosis is likely to contribute to motor neuron vulnerability. Nevertheless, in view of the multifactorial nature of SBMA, the results of this study strongly suggest that endoplasmic reticulum stress is one of the earliest pathological mechanisms occurring in the disease and may therefore represent a potentially useful therapeutic target for the treatment of SBMA.

Evidence of endoplasmic reticulum stress was also detected *in vivo* in spinal cord motor neurons of AR100 mice. Indeed, the expression levels of a number of endoplasmic reticulum stress markers were significantly higher in AR100 spinal cord tissue than in wild-type control tissue, even in presymptomatic mice, as young as 5 days of age, long before the onset of disease symptoms. This observation is similar to that previously reported in *SOD1^G93A^* mice, which model amyotrophic lateral sclerosis (Saxena *et al.*, 2009), and suggests that endoplasmic reticulum stress may be an early event in the pathogenesis of SBMA. Although the relative increase in expression of endoplasmic reticulum stress markers in AR100 spinal cord compared to wild-type control tissue reduced with disease progression, it is likely that this may reflect the progressive decrease in motor neuron survival in AR100 mice as they age.

In conclusion, the results of this study provide evidence of endoplasmic reticulum Ca^2+^ depletion and the existence of Ca^2+^-dependent endoplasmic reticulum stress in cultured embryonic motor neurons from the AR100 mouse model of SBMA, and the presence of endoplasmic reticulum stress in the spinal cord of presymptomatic SBMA mice. The detection of endoplasmic reticulum stress in motor neurons in SBMA mice aged as young as 5 days of age strongly suggests that endoplasmic reticulum stress may play an early, possibly causal role in SBMA disease pathogenesis. Furthermore, the beneficial effects of pharmacological inhibition of endoplasmic reticulum stress in AR100 motor neurons *in vitro* not only support the proposal that endoplasmic reticulum stress plays a direct role in SBMA motor neuron degeneration, but also indicate that pharmacological targeting of endoplasmic reticulum stress during early stages of disease may constitute an effective disease-modifying strategy for the treatment of SBMA.

## Funding

This work was supported by the Institute of Neurology Kennedy’s Disease Research Fund, Motor Neuron Disease Association (MNDA), National Institutes of Health (R01 NS041648 to A.R.L.), and by the Muscular Dystrophy Association (Basic Research Grant to A.R.L.). K.M. was funded by an MRC studentship. The MRC Centre for Neuromuscular Diseases is supported by an MRC Centre Grant. G.S. is supported by Parkinson's UK, Wellcome Trust and Telethon (Italy) AIRC (Italy). L.G. is the Graham Watts Senior Research Fellow, funded by The Brain Research Trust and the European Community's Seventh Framework Programme (FP7/2007-2013).

## Supplementary material

Supplementary material is available at *Brain* online.

Supplementary Data

## Abbreviations

AR100: androgen receptor with pathogenic 100 glutamine repeat
BiP: binding immunoglobulin protein
CHOP: CCAAT/enhancer binding protein-homologous protein
PERK: protein kinase RNA-like endoplasmic reticulum kinase
SBMA: spinal and bulbar muscular atrophy
SERCA: sarcoendoplasmic reticulum Ca^2+^ ATPase

## References

[awu114-B1] Abramov AY, Duchen MR (2008). Mechanisms underlying the loss of mitochondrial membrane potential in glutamate excitotoxicity. Biochim Biophys Acta.

[awu114-B2] Appel SH, Beers D, Siklos L, Engelhardt JI, Mosier DR (2001). Calcium: the Darth Vader of ALS. Amyotroph Lateral Scler Other Motor Neuron Disord.

[awu114-B3] Atkin JD, Farg MA, Walker AK, McLean C, Tomas D, Horne MK (2008). Endoplasmic reticulum stress and induction of the unfolded protein response in human sporadic amyotrophic lateral sclerosis. Neurobiol Dis.

[awu114-B4] Bassik MC, Scorrano L, Oakes SA, Pozzan T, Korsmeyer SJ (2004). Phosphorylation of BCL-2 regulates ER Ca2+ homeostasis and apoptosis. EMBO J.

[awu114-B5] Bilsland LG, Nirmalananthan N, Yip J, Greensmith L, Duchen MR (2008). Expression of mutant SOD1 in astrocytes induces functional deficits in motoneuron mitochondria. J Neurochem.

[awu114-B6] Boyce M, Bryant KF, Jousse C, Long K, Harding HP, Scheuner D (2005). A selective inhibitor of eIF2alpha dephosphorylation protects cells from ER stress. Science.

[awu114-B7] Bravo R, Vicencio JM, Parra V, Troncoso R, Munoz JP, Bui M (2011). Increased ER-mitochondrial coupling promotes mitochondrial respiration and bioenergetics during early phases of ER stress. J Cell Sci.

[awu114-B8] Chami M, Oules B, Szabadkai G, Tacine R, Rizzuto R, Paterlini-Brechot P (2008). Role of SERCA1 truncated isoform in the proapoptotic calcium transfer from ER to mitochondria during ER stress. Mol Cell.

[awu114-B9] Demaurex N, Distelhorst C (2003). Cell biology. Apoptosis–the calcium connection. Science.

[awu114-B10] Duennwald ML, Lindquist S (2008). Impaired ERAD and ER stress are early and specific events in polyglutamine toxicity. Genes Dev.

[awu114-B52] Duong FH, Warter JM, Poindron P, Passilly P (1999). Effect of the nonpeptide neurotrophic compound SR 57746A on the phenotypic survival of purified mouse motoneurons. Br J Pharmacol.

[awu114-B11] Fischbeck KH (2001). Polyglutamine expansion neurodegenerative disease. Brain Res Bull.

[awu114-B53] Fischer LR, Igoudjil A, Magrane J (2011). SOD1 targeted to the mitochondrial intermembrane space prevents motor neuropathy in the Sod1 knockout mouse. Brain.

[awu114-B12] Foradori CD, Handa RJ (2008). Living or dying in three quarter time: neonatal orchestration of hippocampal cell death pathways by androgens and excitatory GABA. Exp Neurol.

[awu114-B54] Fratta P, Malik B, Gray A (2013). FUS is not dysregulated by the spinal bulbar muscular atrophy androgen receptor polyglutamine repeat expansion. Neurobiol Aging.

[awu114-B13] Gatchel JR, Zoghbi HY (2005). Diseases of unstable repeat expansion: mechanisms and common principles. Nat Rev Genet.

[awu114-B14] Harding HP, Zhang Y, Bertolotti A, Zeng H, Ron D (2000). Perk is essential for translational regulation and cell survival during the unfolded protein response. Mol Cell.

[awu114-B15] Heinlein CA, Chang C (2001). Role of chaperones in nuclear translocation and transactivation of steroid receptors. Endocrine.

[awu114-B16] Kakkar P, Awasthi S, Viswanathan PN (1992). Oxidative changes in brain of aniline-exposed rats. Arch Environ Contam Toxicol.

[awu114-B17] Katsuno M, Adachi H, Kume A, Li M, Nakagomi Y, Niwa H (2002). Testosterone reduction prevents phenotypic expression in a transgenic mouse model of spinal and bulbar muscular atrophy. Neuron.

[awu114-B18] Katsuno M, Tanaka F, Adachi H, Banno H, Suzuki K, Watanabe H (2012). Pathogenesis and therapy of spinal and bulbar muscular atrophy (SBMA). Prog Neurobiol.

[awu114-B19] Kaufman RJ (1999). Stress signaling from the lumen of the endoplasmic reticulum: coordination of gene transcriptional and translational controls. Genes Dev.

[awu114-B20] Kessel D (2006). Protection of Bcl-2 by salubrinal. Biochem Biophys Res Commun.

[awu114-B21] La Spada AR, Wilson EM, Lubahn DB, Harding AE, Fischbeck KH (1991). Androgen receptor gene mutations in X-linked spinal and bulbar muscular atrophy. Nature.

[awu114-B22] Lee SK, Kim YS (2013). Phosphorylation of eIF2alpha attenuates statin-induced apoptosis by inhibiting the stabilization and translocation of p53 to the mitochondria. Int J Oncol.

[awu114-B23] Ma Y, Brewer JW, Diehl JA, Hendershot LM (2002). Two distinct stress signaling pathways converge upon the CHOP promoter during the mammalian unfolded protein response. J Mol Biol.

[awu114-B24] Malik B, Currais A, Andres A, Towlson C, Pitsi D, Nunes A (2008). Loss of neuronal cell cycle control as a mechanism of neurodegeneration in the presenilin-1 Alzheimer's disease brain. Cell Cycle.

[awu114-B25] Malik B, Fernandes C, Killick R, Wroe R, Usardi A, Williamson R (2012). Oligomeric amyloid-beta peptide affects the expression of genes involved in steroid and lipid metabolism in primary neurons. Neurochem Int.

[awu114-B26] Malik B, Nirmalananthan N, Bilsland LG, La Spada AR, Hanna MG, Schiavo G (2011). Absence of disturbed axonal transport in spinal and bulbar muscular atrophy. Hum Mol Genet.

[awu114-B27] Malik B, Nirmalananthan N, Gray AL, La Spada AR, Hanna MG, Greensmith L (2013). Co-induction of the heat shock response ameliorates disease progression in a mouse model of human spinal and bulbar muscular atrophy: implications for therapy. Brain.

[awu114-B55] McClive PJ, Sinclair AH (2001). Rapid DNA extraction and PCR-sexing of mouse embryos. Mol Reprod Dev.

[awu114-B28] Maravall M, Mainen ZF, Sabatini BL, Svoboda K (2000). Estimating intracellular calcium concentrations and buffering without wavelength ratioing. Biophys J.

[awu114-B29] Michelangeli F, East JM (2011). A diversity of SERCA Ca2+ pump inhibitors. Biochem Soc Trans.

[awu114-B30] Nakagawa T, Yuan J (2000). Cross-talk between two cysteine protease families. Activation of caspase-12 by calpain in apoptosis. J Cell Biol.

[awu114-B31] Nihei Y, Ito D, Okada Y, Akamatsu W, Yagi T, Yoshizaki T (2013). Enhanced aggregation of androgen receptor in induced pluripotent stem cell-derived neurons from spinal and bulbar muscular atrophy. J Biol Chem.

[awu114-B32] Parekh AB, Penner R (1997). Store depletion and calcium influx. Physiol Rev.

[awu114-B33] Paschen W, Doutheil J (1999). Disturbance of endoplasmic reticulum functions: a key mechanism underlying cell damage?. Acta Neurochir Suppl.

[awu114-B34] Perutz MF, Johnson T, Suzuki M, Finch JT (1994). Glutamine repeats as polar zippers: their possible role in inherited neurodegenerative diseases. Proc Natl Acad Sci USA.

[awu114-B35] Reijonen S, Putkonen N, Norremolle A, Lindholm D, Korhonen L (2008). Inhibition of endoplasmic reticulum stress counteracts neuronal cell death and protein aggregation caused by N-terminal mutant huntingtin proteins. Exp Cell Res.

[awu114-B36] Rocchi A, Pennuto M (2013). New routes to therapy for spinal and bulbar muscular atrophy. J Mol Neurosci.

[awu114-B37] Ross CA (2002). Polyglutamine pathogenesis: emergence of unifying mechanisms for Huntington's disease and related disorders. Neuron.

[awu114-B38] Saxena S, Cabuy E, Caroni P (2009). A role for motoneuron subtype-selective ER stress in disease manifestations of FALS mice. Nat Neurosci.

[awu114-B39] Smani T, Zakharov SI, Csutora P, Leno E, Trepakova ES, Bolotina VM (2004). A novel mechanism for the store-operated calcium influx pathway. Nat Cell Biol.

[awu114-B40] Soo KY, Atkin JD, Farg M, Walker AK, Horne MK, Nagley P (2012). Bim links ER stress and apoptosis in cells expressing mutant SOD1 associated with amyotrophic lateral sclerosis. PLoS One.

[awu114-B41] Sopher BL, Thomas PS, LaFevre-Bernt MA, Holm IE, Wilke SA, Ware CB (2004). Androgen receptor YAC transgenic mice recapitulate SBMA motor neuronopathy and implicate VEGF164 in the motor neuron degeneration. Neuron.

[awu114-B42] Stenoien DL, Cummings CJ, Adams HP, Mancini MG, Patel K, DeMartino GN (1999). Polyglutamine-expanded androgen receptors form aggregates that sequester heat shock proteins, proteasome components and SRC-1, and are suppressed by the HDJ-2 chaperone. Hum Mol Genet.

[awu114-B43] Szegezdi E, FitzGerald U, Samali A (2003). Caspase-12 and ER-stress-mediated apoptosis: the story so far. Ann N Y Acad Sci.

[awu114-B44] Szegezdi E, Logue SE, Gorman AM, Samali A (2006). Mediators of endoplasmic reticulum stress-induced apoptosis. EMBO Rep.

[awu114-B45] Takeyama K, Ito S, Yamamoto A, Tanimoto H, Furutani T, Kanuka H (2002). Androgen-dependent neurodegeneration by polyglutamine-expanded human androgen receptor in Drosophila. Neuron.

[awu114-B46] Thomas M, Yu Z, Dadgar N, Varambally S, Yu J, Chinnaiyan AM (2005). The unfolded protein response modulates toxicity of the expanded glutamine androgen receptor. J Biol Chem.

[awu114-B47] Thomas PS, Fraley GS, Damien V, Woodke LB, Zapata F, Sopher BL (2006). Loss of endogenous androgen receptor protein accelerates motor neuron degeneration and accentuates androgen insensitivity in a mouse model of X-linked spinal and bulbar muscular atrophy. Hum Mol Genet.

[awu114-B48] Walcott JL, Merry DE (2002). Ligand promotes intranuclear inclusions in a novel cell model of spinal and bulbar muscular atrophy. J Biol Chem.

[awu114-B49] Williams AJ, Paulson HL (2008). Polyglutamine neurodegeneration: protein misfolding revisited. Trends Neurosci.

[awu114-B50] Yoneda T, Imaizumi K, Oono K, Yui D, Gomi F, Katayama T (2001). Activation of caspase-12, an endoplastic reticulum (ER) resident caspase, through tumor necrosis factor receptor-associated factor 2-dependent mechanism in response to the ER stress. J Biol Chem.

[awu114-B51] Yu Z, Wang AM, Adachi H, Katsuno M, Sobue G, Yue Z (2011). Macroautophagy is regulated by the UPR-mediator CHOP and accentuates the phenotype of SBMA mice. PLoS Genet.

