# Supplementary material for: Endoplasmic reticulum stress in spinal and bulbar muscular atrophy: a potential target for therapy
Source: Brain. 2014 Jun 4;137(7):1894–906. doi: 10.1093/brain/awu114 (PMC4065020; doi:10.1093/brain/awu114)
Supplement: Supplementary Data [file supp_137_7_1894__index.html]

Endoplasmic reticulum stress in spinal and bulbar muscular atrophy: a potential target for therapy — Supplementary Data 

# Endoplasmic reticulum stress in spinal and bulbar muscular atrophy: a potential target for therapy

## Supplementary Data

files

**Files in this Data Supplement:**

- Supplementary Data - doc file
