# Supplementary material for: Endoplasmic reticulum stress in spinal and bulbar muscular atrophy: a potential target for therapy
Source: Brain. 2014 Jun 4;137(7):1894–906. doi: 10.1093/brain/awu114 (PMC4065020; doi:10.1093/brain/awu114)
Supplement: Supplementary Data [file supp_awu114_brain-2013-01938-File009.doc]

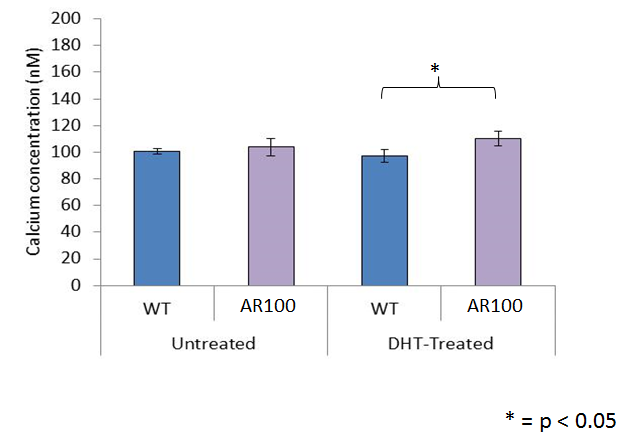


**Supplementary Figure 1. AR100 motor neurons have the highest level of basal cytosolic calcium under calcium-free recording conditions.**

When basal cytosolic calcium is measured in a calcium-free recording medium, it appears that DHT- treated AR100 motor neurons have the highest level of basal cytosolic calcium, which is significantly higher than that observed in treated DHT-WT motor neurons (*p <0.05). However, there is little difference between the untreated WT and AR100 motor neurons.


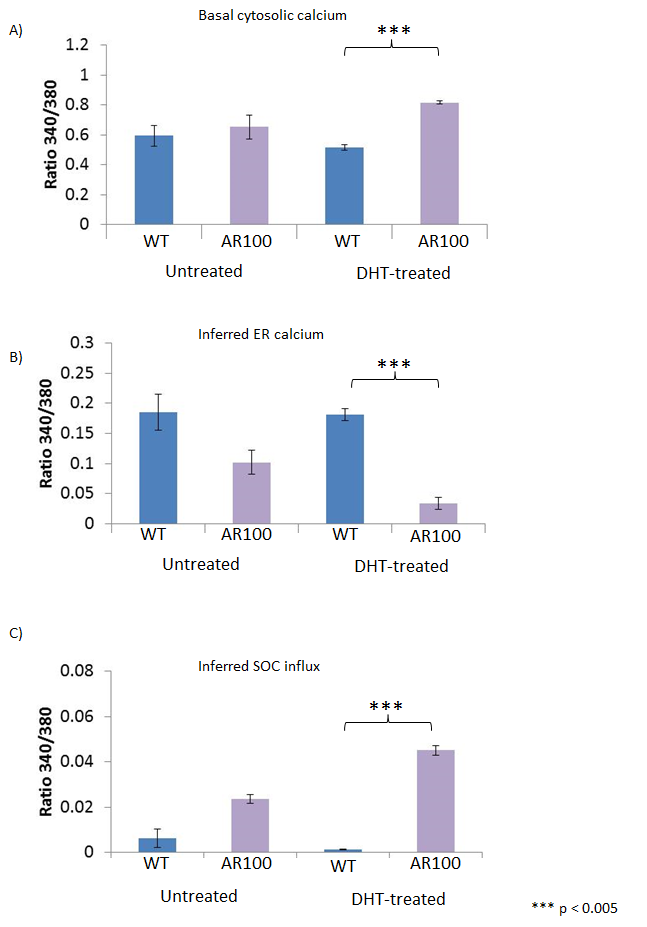


**Supplementary Figure 2. The trend in changes in cytosolic calcium observed using Fluo 4-AM is reproduced when Fura 2 is employed as the calcium indicator.**

(A) Basal cytosolic calcium is highest in DHT-treated AR100 motor neurons and lowest in DHT-treated WT motor neurons. This difference is significant (p < 0.005). (B) Inferred ER calcium is lowest in DHT-treated AR100 motor neurons and highest in DHT-treated WT motor neurons. As is the case when Fluo 4-AM is used, the difference is significant (p < 0.005). (C) Finally, the trend observed when measuring SOC influx using Fluo 4-AM is reproduced with Fura 2: treated AR100 motor neurons display the highest level of SOC influx whilst treated WT motor neurons display the lowest level.


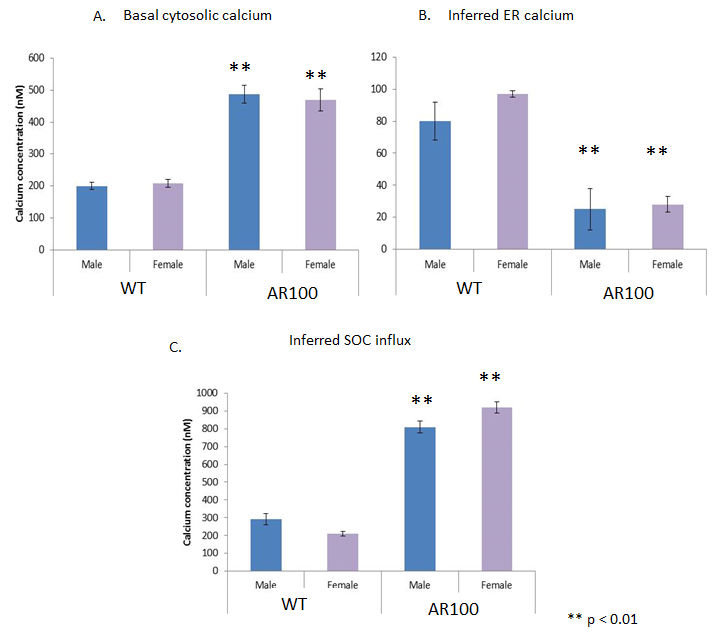


**Supplementary Figure 3**. **Negligible difference in Ca^2+^ handling between male and female motor neurons**

There is a negligible difference between male and female motor neurons of the same genotype with respect to all three parameters measured, but a significant difference between motor neurons of the same sex but different genotype


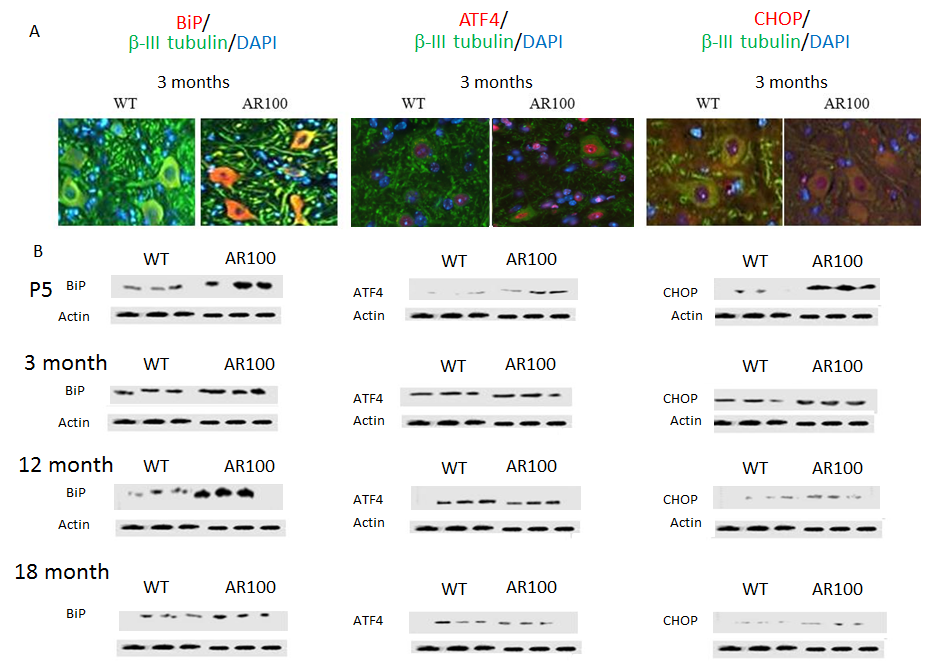


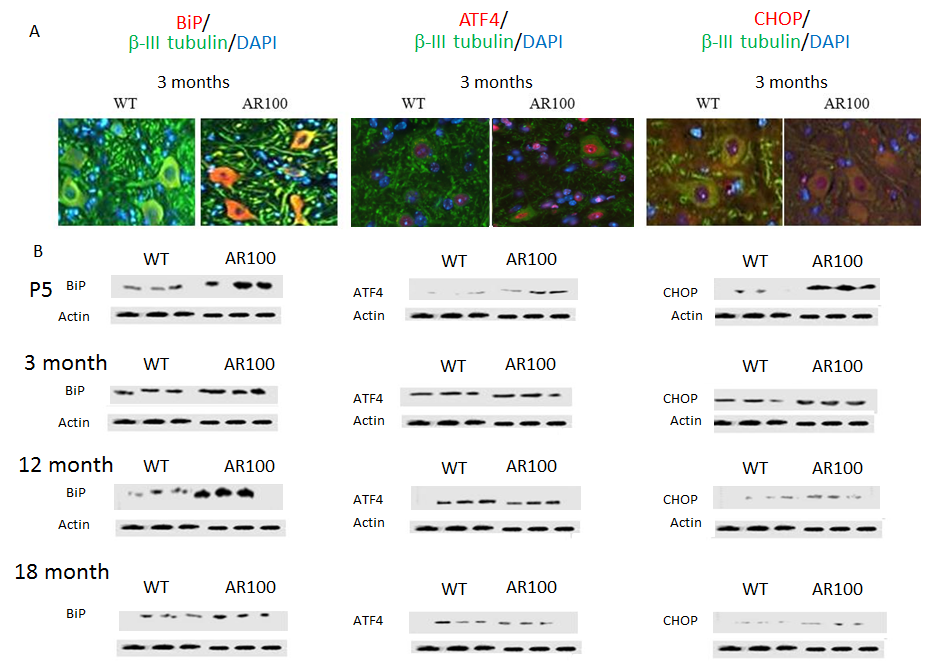


**Supplementary Figure 4. ER stress markers are elevated in spinal cord of spinal and bulbar muscular atrophy mice at different stages of the disease.**
